# Beyond Household Adversities: Synthetic Imputation of Emotional, Physical and Sexual Abuse for Children Aged <18 Years in the National Survey on Children’s Health (NSCH) Through Parameter Bridging from the Behavioral Risk Factor Surveillance System (BRFSS) and Youth Risk Behavior Surveillance System (YRBSS)

**DOI:** 10.3390/bs16091652

**Published:** 2026-09-15

**Authors:** David Brown, Robert F. Anda

**Affiliations:** 1 School of Public and Community Medicine, University of Montana, Missoula, MT 59812, USA; robanda@bellsouth.net; 2 Managing Principle, BCGI LLC/pivot-23.5°, Cornelius, NC 28031, USA; 3 ACE Interface LLC, Peachtree City, GA 30269, USA

**Keywords:** adverse childhood experiences, construct validity, public health surveillance, models, statistical

## Abstract

The National Survey of Children’s Health (NSCH) is widely used to estimate adverse childhood experiences (ACEs) in pediatric populations, but it only captures household adversity and economic hardship, omitting direct measures of emotional, physical, and sexual abuse present in the original ACE Study. We quantified this measurement gap by projecting unmeasured abuse domains onto the pooled 2023–2024 NSCH (n = 104,838) using parameter-bridging models estimated from the Behavioral Risk Factor Surveillance System (BRFSS; adults aged 18–49 years, n = 27,499) and the Youth Risk Behavior Surveillance System (YRBSS; adolescents, n = 11,016), both of which directly measure these abuse domains. Raw NSCH data classify 93.9% of infants under 12 months and 61.4% of adolescents aged 14–17 years as free of any ACEs; incorporating projected abuse domains reduces these figures to approximately 53–57% and 36–46%, respectively, depending on donor source. Conversely, the proportion with four or more ACEs—0.2% and 3.8% in the raw NSCH data for these two age groups—rises to approximately 2% and 7–14% once projected abuse is incorporated. These projected values represent model-based structural risk conditional on a child’s observed household environment, not direct measures of accumulated exposure or observed prevalence. Out-of-sample calibration testing indicates that projections from each donor source carry substantial asymmetric uncertainty: BRFSS-derived estimates over-predict and YRBSS-derived estimates under-predict emotional and sexual abuse relative to each source’s own directly observed rates. Despite this uncertainty, the direction and scale of the gap are consistent across donor sources and sensitivity analyses. NSCH-based ACE indices that omit direct abuse likely substantially understate true childhood adversity, with implications for screening, resource allocation, and cross-population comparisons relying on this widely used pediatric surveillance tool.

## 1. Introduction

Adverse childhood experiences (ACEs) are potentially traumatic events occurring before age 18 years—a developmental window spanning from the prenatal period through adolescence characterized by rapid neuroplasticity (Cross et al., 2017). Chronic exposure to adversity can lead to the sustained activation of the biological stress response, resulting in a prolonged elevation of stress hormones (Danese & McEwen, 2012). Such dysregulation during critical periods can disrupt the development of brain regions essential for self-regulation and executive function, leaving lasting imprints on brain structure and lifelong health (McEwen & Morrison, 2013; McLaughlin et al., 2014). Furthermore, emerging evidence suggests that epigenetic mechanisms may play a significant role in the intergenerational transmission of these early adversity effects (McKenna et al., 2023; Zhou & Ryan, 2023).

The seminal Centers for Disease Control and Prevention-Kaiser Permanente ACE Study categorized ACEs into three domains: abuse (physical, emotional, and sexual), neglect (physical and emotional), and household dysfunction (substance use, mental illness, incarceration, parental separation or divorce, and domestic violence; operationalized in this paper as *household adversities*) (Felitti et al., 1998). These ACEs were originally summarized using a cumulative score reflecting the clinical observation that ACEs tend to co-occur (Dong et al., 2004) and exhibit a dose–response relationship with a broad range of short- and long-term health and social outcomes (Anda et al., 2006). A substantial body of research has since linked higher ACE scores to chronic disease, mental health disorders, and health-risk behaviors, as well as increased healthcare utilization and premature mortality (Anda et al., 2008; Brown et al., 2009; Hughes et al., 2017). Beyond health, ACEs significantly impair educational attainment, employment stability, and social functioning while increasing the likelihood of justice system involvement (Hansen et al., 2021; Houtepen et al., 2020; Huei-Jong Graf et al., 2021; Tzouvara et al., 2023).

Given that ACE-related outcomes unfold across the life course, systematic population-level monitoring is fundamental to guiding prevention and policy. In the United States, two primary surveillance platforms—the Behavioral Risk Factor Surveillance System (BRFSS) (CDC, 2026a) and the Youth Risk Behavior Surveillance System (YRBSS) (CDC, 2026c)—serve as the foundational tools for monitoring these trends. The BRFSS has collected retrospective ACE data from adults since 2009, capturing all three domains through modules derived from the original ACE Study. More recently, the 2023 YRBSS expanded this monitoring to adolescent populations, utilizing cognitively tested questions (Massey & Virkar, 2022) adapted to capture contemporaneous and retrospective experiences of abuse, neglect, and household adversities among high school students. Together, these systems provide a comprehensive self-reported longitudinal view of the impacts of adversity from adolescence through late adulthood.

While the BRFSS and YRBSS offer a direct window into the full spectrum of self-reported adversity during adolescence and adulthood, the National Survey of Children’s Health (NSCH) (HRSA, 2026) is an important tool to monitor the health and well-being of children from birth through age 17 years. As a nationally representative survey of parents and guardians, the NSCH structural framework differs from BRFSS and YRBSS in two critical ways. First, unlike the self-reported measures found in adult and adolescent surveys, NSCH data are reported by proxy. Second, and more importantly for surveillance of exposure to ACEs, the NSCH only captures a subset of the original ACE Study domains—including household adversities and neglect while excluding direct measures of emotional, physical, and sexual abuse. While this design effectively maps household-level socio-environmental dysfunction-related risk, it structurally truncates the multi-domain matrix required for a complete ACE score framework. Applying the broad label ‘ACEs’ to these partial indices creates a misleading equivalence with that of the original ACE Study, compromising comparability across population studies. This measurement gap is not merely definitional. A substantial synthesis literature links cumulative childhood adversity to elevated risks of adolescent violence perpetration and victimization, underscoring why omitting direct abuse domains from a pediatric surveillance index has consequences beyond measurement precision (Hughes et al., 2017; Norman et al., 2012).

Consequently, the purpose of this analysis is two-fold. First, we evaluated the construct completeness of the current approach used to assess cumulative childhood adversity by quantifying the empirical magnitude of this measurement gap within the NSCH. And second, we attempted to reconstruct the foundational multi-domain structure of the ACE framework using a donor–recipient parameter bridging approach. By projecting parameters from self-reported surveillance systems (BRFSS/YRBSS) onto observed pediatric household adversity patterns, we aim to demonstrate how the truncation of core abuse domains fundamentally alters the operational validity of the NSCH-derived ACE score metric.

## 2. Methods

We utilized a multi-survey donor–recipient framework to estimate unmeasured abuse domains within the NSCH. A detailed description of the NSCH methods is available elsewhere (US Census Bureau, 2023, 2024). The recipient sample consisted of 104,838 (out of an overall n = 106,537) children aged 0–17 years from the pooled 2023–2024 NSCH with non-missing information on age, sex, race/ethnicity and ACE components. Donor parameters were derived from the pooled 2023–2024 BRFSS ACE module, restricted to adults aged 18–49 years (to ensure age-period consistency) with non-missing information on sex, race/ethnicity and ACE components (n = 27,499 of 88,199 observations from the 14 states that included the optional ACE module; see Supplementary Table S1), and the 2023 YRBSS ACE module (n = 11,016 of a total 20,103 observations). Analyses were completed among those observations with complete information on sex, race/ethnicity and ACE components. We observed no meaningful differences in sex or race/ethnicity between those with complete and missing data on ACE components.

Because donor data were utilized to examine structural relationships assumed to be temporally stable over the study period, rather than to estimate annual population prevalence, we applied a constant sample contribution approach in normalizing analytic weights for the BRFSS. We maintained the integrity of the complex sampling frame by nesting primary sampling units (PSUs) and strata within survey years prior to pooling. Sampling weights were then normalized within each annual cycle by dividing the original final weight by the year-specific sum of weights, and a final pooled weight was then constructed by scaling these normalized values by the total aggregate sample size across the study period. For the recipient NSCH data, annual sampling weights were divided by *k* = 2 to generate point estimates and population totals representative of the average annual population over the two years of survey data.

Structural and semantic harmonization was performed across all three datasets to align variables for household adversities (substance use, mental illness, incarceration, domestic violence, and parental divorce) and economic hardship (neglect). (N.B.: Regarding the latter, it is important to note that the NSCH asks about a family’s economic circumstance rather than about caregiver behavior-related neglect, which is the focus of the original ACE Study physical neglect construct. To remain consistent with the original ACE Study, we have chosen to use the term economic hardship throughout this paper). This process ensured that the predictive stressors used in the donor models were consistent with the observed markers in the recipient survey. Detailed survey question wording for all measured ACEs across the three survey platforms are provided in Table 1.

Initial analyses quantified the design-based bivariate associations between each of the ACE categories measured in the BRFSS and YRBSS (Table 2a,b). We calculated conditional probabilities, P(Abuse|Household Adversity), to evaluate the prevalence of abuse within specific household environments. These comparisons allowed for an assessment of the predictive weight of infrequent indicators and the characteristic co-occurrence of stressors, such as the overlap between domestic violence and physical abuse. Adolescent patterns were analyzed within the context of the truncated exposure window inherent in concurrent reporting (reflecting incomplete exposure relative to potential peak-risk developmental periods), explicitly contrasting emergent risk in youth with the cumulative lifetime burden captured in adult retrospective recall.

Multivariate logistic regression donor models were estimated within the BRFSS and YRBSS to extract parameter estimates ($\hat{\beta }$) and their associated variance–covariance matrices ($\hat{V}$) for parameter projection. Separate models were specified for each abuse domain (emotional, physical, and sexual). Predictors included the five household adversity domains and economic hardship (neglect). We also specified reduced BRFSS models that omitted the parental divorce marker to maintain parity with the YRBSS, where that specific marker was not available for modeling. All donor models were adjusted for respondent sex, and race/ethnicity. Age was evaluated but excluded from the final donor models. There is no principled value of a BRFSS 5-year adult age category (e.g., ages 30–34) or a YRBSS adolescent age category to assign to a NSCH child whose age falls outside the donor sample’s range entirely, so rather than assign an arbitrary or extrapolated value, age was dropped as a covariate while sex and race/ethnicity, which carry a coherent shared meaning across all three surveys, were retained.

### Synthetic Imputation and Parameter Bridging

Estimated parameters from the BRFSS/YRBSS models were projected onto the observed household patterns in the pooled NSCH using a stochastic simulation-based imputation approach. To satisfy Rubin’s rules for variance estimation and ensure stable prevalence estimates, we generated 50 imputation replicates (M = 50). For each child in the NSCH, a linear predictor was calculated by drawing a random vector of coefficients from the donor’s posterior distribution: $\tilde{\beta_{m}}$ $\sim N(\hat{\beta ,}\hat{V})$.

Stochastic draws from the resulting inverse-logit probabilities were used to assign binary values for each unmeasured abuse domain. These imputed values were then summed with the observed NSCH household markers to construct composite ACE scores (categorized as 0, 1, 2, 3, and 4+). Stata 14 (Stata Corp., College Station, TX, USA) was used to conduct all analyses to account for the complex survey design of each survey and the added uncertainty inherent in the synthetic projection process in the last steps. Final projected probabilities and 95% simulation intervals, computed separately for each age band via the multiple imputation (mi) estimate command, are reported by age group throughout; as discussed below, these age band-specific values represent projected structural risk rather than age-specific realized prevalence.

The validity of these synthetic estimates rests on the applicability of coefficients derived from the donor surveys to the children sampled in the NSCH, as well as reporting consistency across survey platforms. A primary assumption of this framework is that the structural relationship between household adversity and abuse (emotional, physical, and sexual) in the recipient NSCH sample mirrors the patterns observed in the donor datasets. To mitigate potential cohort effects and account for secular trends in the distribution and co-occurrence of childhood trauma, we utilized the most recent available cycles of the BRFSS ACE module. Furthermore, we acknowledge the inherent differences in reporting modalities across the three platforms. While the NSCH relies on proxy reports from parents or guardians regarding household stressors, the donor datasets utilize self-reports (retrospective in the BRFSS and concurrent in the YRBSS).

The inclusion of YRBSS data serves as a critical comparison in this analysis. By examining adolescent self-reports, we were able to evaluate whether the associations between household adversity and abuse significantly differed from those captured in the adult retrospective recall, thereby providing a sensitivity check for the stability of the donor parameters across different reporters and developmental stages. We also acknowledge the assumption of measurement equivalence across surveys; despite rigorous semantic harmonization, differences in survey modality and respondent interpretation may impact the threshold for reporting household stressors (i.e., the determination of reporting mental illness in the household may differ). Furthermore, our models assume that the structural parameters derived from the donor data are not significantly confounded by variables unobserved in the recipient dataset. Finally, while our stochastic simulation accounts for the statistical uncertainty of the donor parameters, it assumes a stable covariance structure between latent abuse domains across different surveillance populations.

To assess whether parameter estimates derived from the adult BRFSS sample transport to a life stage and reporting context closer to the pediatric target population, we conducted an out-of-sample calibration analysis using the YRBSS, in which all three abuse domains are directly self-reported. The reduced BRFSS donor models (excluding divorce/separation, to match covariates available in both surveys) were applied to YRBSS respondents’ own household adversity and neglect indicators to generate a predicted probability of each abuse domain. These predicted probabilities were compared to YRBSS respondents’ own directly reported abuse status using calibration-in-the-large and a calibration slope (both estimated using the YRBSS survey design), and discrimination (the C-statistic, computed without survey weights due to software constraints; this is interpreted as an approximate signal of relative discrimination rather than a design-based population estimate).

To determine whether this calibration pattern was specific to the direction of projection, the test was repeated in reverse—applying the reduced YRBSS donor models to BRFSS respondents’ own indicators and comparing to BRFSS respondents’ own directly reported abuse status. Both directions were then repeated using the chained donor models (see below), substituting each population’s own observed emotional and physical abuse statuses for the chain-input covariates to determine whether the calibration pattern was sensitive to the domain-independence assumption.

As a sensitivity analysis addressing concerns about the NSCH economic hardship item’s construct validity relative to the donor surveys’ caregiver effort measure of neglect (see Discussion), all nine chained donor models were re-estimated with this item removed entirely from the covariate set, and the item was correspondingly dropped from the composite ACE score (yielding an 8-item score for the BRFSS Full specification and a 7-item scores for BRFSS Reduced and YRBSS in place of the 9- and 8-item primary specifications). All other aspects of model specification, the chained M = 50 stochastic projection procedure, and survey design estimation were unchanged from the primary analysis. Results are reported in Supplementary Table S2.

Because the donor coefficient vectors are estimated in an external sample (BRFSS or YRBSS) and applied to a structurally different recipient sample (NSCH) whose true abuse status is unobserved, this procedure does not satisfy the conditions under which Rubin’s rules for multiple imputation guarantee valid inference. The model used to generate the projected values is not the same as, and was not built for, the population it is being applied to, so the reported simulation intervals reflect donor parameter and Bernoulli draw variance only; they do not reflect the full uncertainty a matched, purpose-built imputation procedure would capture.

## 3. Results

Differences were observed in the crude prevalence of stressors across adolescents, adult self-reports and adult proxy reports for their children (Table 1). In general, prevalence values tended to be lowest for adult proxy reports with one notable exception: reported neglect was higher in NSCH (14.3%) than in either donor survey (8.1–8.3%), consistent with the construct differences between the NSCH’s economic hardship item and the donor surveys’ caregiver effort item discussed below. Values of divorce or separation in the household were meaningfully higher among adult self-reports than adult proxy reports. Values of mental illness in the household were similar for adolescents and adult self-reports but much higher compared to adult proxy reports. Differences were also observed for household substance abuse, violence, and incarceration.

Conditional probability matrices indicate that individual ACEs rarely occur in isolation, with specific ACE categories carrying significant predictive weight for additional adversity domains (Table 2a,b). Among adults aged 18–49 years, household substance abuse and domestic violence serve as the primary anchors for multi-domain exposure. For those reporting household substance abuse, there is an 82% probability of experiencing emotional abuse and a 71% probability of co-occurring mental illness in the home. Similarly, household violence is strongly associated with direct victimization, with a 68% conditional probability of physical abuse and an 80% probability of emotional abuse. In the adolescent sample, household violence remains the strongest predictor of direct physical victimization, with a 71% conditional probability. Adolescents living with household incarceration report a 67% probability of concurrent substance abuse and a 56% probability of mental illness within the household. Sexual abuse in this population is highly clustered with emotional maltreatment, carrying a 72% conditional probability.

For both adults (BRFSS) and adolescents (YRBSS), approximately one-third of individuals living in households with no forms of household adversity markers still experienced at least one form of abuse, indicating a substantial hidden basal burden that the household adversity markers alone do not capture (Table 3a,b). A distinct discrepancy exists in the reporting of all three forms of abuse between the two populations. Adults identified a much higher density of multiple abuse forms than adolescents, perhaps reflecting the fact that the peak exposure window for some forms of abuse among adolescents may be truncated depending on the respondent’s age at the time of interview and/or reflect a recognition lag such that adults have had the benefit of longer processing to identify and name experiences as abuse. For both adults and adolescents, we observed a dramatic increase in the probability of abuse as household adversity stressors accumulate. In the BRFSS, the probability of experiencing at least one form of abuse rises from 34% among those with zero household adversity markers to 96% among those reporting all five markers. A similar trajectory was observed for adolescents.

Projected probabilities of emotional, physical, and sexual abuse across the pediatric population (0–17 years) using the bridged parameter estimates from the donor models reveals significant variance depending on the donor source used and a child’s age in the NSCH data. We observed remarkable consistency between the full (five household adversity stressors + neglect) and reduced (four household adversity markers + neglect, excluding divorce) BRFSS models (Table 4). Across all three abuse domains, the estimates derived from these two models rarely differ by more than a 1 to 2 percentage points, suggesting that the structural relationship between the remaining household stressors and abuse is not significantly altered by the omission of the divorce/separation stressor. The BRFSS parameters consistently yielded higher projected probabilities for emotional and sexual abuse than the YRBSS parameters. This is most acute in sexual abuse, where the adult-recalled parameters project a probability more than double that of the adolescent-reported parameters by age 14–17 years (12.6% vs. 4.7%). For physical abuse, the YRBSS donor source projected a higher burden in children under age 5 years than the BRFSS. For example, in the 1–2 year old age group, YRBSS projects 20.9% physical abuse compared to 18.7% from the BRFSS full model.

Given this divergence between donor sources, we assessed the transportability of BRFSS-derived parameter estimates directly, since no external benchmark exists for the true prevalence of unmeasured abuse domains within the NSCH itself. Using the one population in which ground truth is available—YRBSS respondents’ own directly self-reported abuse status—we applied the reduced BRFSS donor models to YRBSS covariates and evaluated calibration-in-the-large, a calibration slope, and discrimination (C-statistic). This out-of-sample calibration analysis revealed meaningful miscalibration for two of the three abuse domains. For emotional abuse, the model over-predicted average risk relative to YRBSS respondents’ own reports (predicted mean 45.9%, 95% CI 44.5–47.3, versus observed 34.5%, 95% CI 32.6–36.4) and showed evidence of overdispersion (calibration slope 0.68, 95% CI 0.60–0.76, significantly below the ideal value of 1.0). For sexual abuse, the model substantially over-predicted the average level (predicted 16.9%, 95% CI 16.1–17.8, versus observed 7.1%, 95% CI 6.1–8.0, or approximately 2.4 times the observed rate), though the relative ranking of risk was well-calibrated (slope 1.00, 95% CI 0.89–1.11), indicating the miscalibration here is primarily a shift in overall level rather than a distortion of relative risk. Physical abuse showed the closest correspondence between predicted and observed values (29.8%, 95% CI 29.0–30.6, versus 32.1%, 95% CI 29.9–34.3), though its calibration slope (0.85, 95% CI 0.78–0.92) indicates modest overdispersion. Discrimination was acceptable to good across all three domains (C-statistic: emotional 0.72, physical 0.73, sexual 0.79), indicating the donor models rank-order relative risk reasonably well even where absolute calibration is imperfect.

To determine whether this pattern was specific to the direction of projection or reflected a broader difference between the two source populations, we conducted the same test in reverse, applying the reduced YRBSS donor models to BRFSS respondents’ own household adversity and neglect markers and comparing the resulting predictions to BRFSS respondents’ own directly reported abuse status. The result was symmetric. YRBSS-derived predictions under-predicted emotional abuse relative to BRFSS respondents’ own reports (predicted mean 33.3%, 95% CI 33.0–33.7, versus observed 45.0%, 95% CI 44.0–46.1) and substantially under-predicted sexual abuse (predicted 6.6%, 95% CI 6.4–6.7, versus observed 16.7%, 95% CI 16.0–17.5 or less than half the observed rate) while showing comparatively close correspondence for physical abuse (predicted 31.7%, 95% CI 31.3–32.1, versus observed 29.8%, 95% CI 28.9–30.8). Calibration slopes in this direction were close to the ideal value of 1.0 (emotional 1.08, 95% CI 1.02–1.13; physical 1.05, 95% CI 1.00–1.10; sexual 0.90, 95% CI 0.84–0.95), and discrimination was similar to the reverse direction (C-statistics: emotional 0.69, physical 0.71, sexual 0.73).

We then tested whether this calibration pattern was sensitive to the domain-independence assumption and repeated both directions of the calibration test using the chained donor models, substituting each population’s own observed emotional and physical abuse statuses for the chain-input covariates. For physical abuse, chaining improved discrimination in both directions (BRFSS→YRBSS: C-statistic 0.81, 95% CI 0.80–0.81, versus 0.73 in the marginal model; YRBSS→BRFSS: 0.78, 95% CI 0.77–0.78, versus 0.71) but widened the calibration-in-the-large gap in both directions (BRFSS→YRBSS: predicted 25.6–27.6% versus observed 32.1%, 95% CI 29.9–34.3%, a wider gap than the marginal model’s 29.0–30.6% versus the same observed value; YRBSS→BRFSS: predicted 35.6–36.7% versus observed 29.8%, 95% CI 28.9–30.8%, again wider than the marginal model’s 31.3–32.1%). Calibration slopes for physical abuse remained close to the ideal value of 1.0 in both directions (0.98, 95% CI 0.92–1.05, BRFSS→YRBSS; 0.92, 95% CI 0.88–0.96, YRBSS→BRFSS). For sexual abuse, the chained models closely reproduced the marginal models’ findings: BRFSS→YRBSS predicted 15.5–17.4% versus observed 7.1% (95% CI 6.1–8.0%), and YRBSS→BRFSS predicted 6.9–7.2% versus observed 16.7% (95% CI 15.9–17.5%)—both consistent with the approximately 2.3-fold over-prediction and less-than-half under-prediction already reported for the marginal models.

The integration of unmeasured abuse domains into the NSCH framework reveals a contrast between the raw NSCH measurement of accumulated childhood adversity and the estimated total ACE burden. The bridged parameter values indicate that the raw NSCH adversity score, derived solely from household adversity and neglect, substantially overestimates the proportion of children with no adversity exposure while significantly masking the prevalence of high-burden (4+ ACEs) profiles (Table 5). In fact, for infants under 12 months, the raw NSCH data suggests that more than 90% of the population is ACE-free. However, our calibration analysis (see above) found that projections from both donor sources carry demonstrated absolute-level uncertainty for emotional and sexual abuse—BRFSS-derived predictions over-predict these domains when tested against YRBSS’s own reports, and YRBSS-derived predictions under-predict them when tested against BRFSS’s own reports—so the BRFSS-derived (49%) and YRBSS-derived (51%) figures reported here should be read as evidence of a substantial reduction in the ACE-free population rather than as precise estimates of its magnitude. As a reminder, it is important to note that these values represent a projection of baseline structural risk based on current household environmental configurations, rather than direct measures of accumulated exposure by a certain age. By ages 14–17 years, the proportion of children with zero ACEs in the raw data stands around 61%. Accounting for unmeasured abuse projects this closer to 33% (BRFSS Full) or 42% (YRBSS). Given the calibration uncertainty documented above, this range is presented as evidence of a substantial reduction rather than a precise pair of bounds.

A sensitivity analysis removing the economic hardship item entirely (Supplementary Table S2) showed a domain-specific pattern. In the BRFSS-derived models, removing the item lowered projected emotional abuse probabilities by 1.5–2.2 percentage points, physical abuse by 2.0–2.5 points, and sexual abuse by 0.5–1.0 points across age categories and both BRFSS specifications, indicating that the item may carry real, independent predictive value in the BRFSS donor sample. In the YRBSS-derived models, the same removal had negligible effect (emotional abuse shifted by at most 0.3 percentage points; physical abuse by at most 0.7 points; several age bands were unchanged to one decimal place), consistent with the item’s non-significant coefficient in the YRBSS donor models (Supplementary Table S3). Composite zero-ACE and 4+-ACE figures shifted more substantially with the item removed. For example, projected zero-ACE probability among infants rose from 53.4% to 60.8% (BRFSS Full) and from 56.6% to 64.9% (YRBSS).

## 4. Discussion

This analysis highlights a fundamental measurement disconnect between the operational definition of ACEs within the National Survey of Children’s Health and the foundational epidemiological construct established by Felitti et al. (1998) in The ACE Study. The issue is not the construct validity of the household adversity indicators captured by the NSCH, which provides invaluable data on household-level socio-environmental risk. Rather, the limitation lies in the systematic omission of direct emotional, physical, and sexual abuse from the survey questionnaire. Interestingly, the 2019 National Health Interview Survey (NHIS) (CDC, 2026b) similarly included questions on childhood household adversity and excluded questions on childhood abuse. The classic paradigm of ACEs rests on the assumption that a comprehensive ACE score requires a minimum foundational base of measurement that explicitly and simultaneously captures the triad of direct abuse, neglect, and household (or home environment) adversity during childhood. When this core matrix is truncated by entirely excluding the abuse domains, the structural integrity of the underlying construct is compromised. Consequently, indices derived solely from the NSCH’s household markers—though labelled as “ACE scores” in recent pediatric literature (Bethell et al., 2014; Goldstein et al., 2021)—fundamentally misquantify exposure to ACEs, introducing a systemic conservative bias that distorts the population-level distribution of childhood adversity. By treating household adversity stressors as a complete proxy for multi-domain childhood adversity, current surveillance frameworks artificially inflate the size of the unexposed cohort while obscuring the true density of high-burden profiles.

A key finding of this cross-survey bridging is the substantial hidden baseline burden of maltreatment occurring outside the context of overt household instability. The design-based bivariate analyses show that approximately one-third of individuals living in environments with zero observed household adversity markers still experienced at least one form of direct abuse. This structural disconnect indicates that traditional household-level markers—such as parental incarceration, substance use, or divorce—function as highly specific but poorly sensitive indicators of a child’s risk. Direct emotional, physical, and sexual abuse can manifest independently of visible family breakdown. As a result, current pediatric surveillance models leveraging NSCH data may systematically overlook a vulnerable segment of the population whose childhood adversity occurs in otherwise structurally stable environments.

### 4.1. Methodological Limitations

Because a biological ground truth for these unobserved domains cannot be independently established within the NSCH itself, we tested calibration in both directions, and against both the marginal donor models and the chained models underlying the final projections reported in Table 4 and Table 5, rather than relying on a single comparison. The pattern for sexual abuse was symmetric and unaffected by chaining: BRFSS-derived predictions over-predict sexual abuse by approximately 2.3-fold when scored against YRBSS respondents’ own reports, and YRBSS-derived predictions under-predict sexual abuse to less than half when scored against BRFSS respondents’ own reports in both the marginal and chained models. Emotional abuse showed a comparable symmetric pattern in the marginal models. Because this miscalibration reverses direction along with the direction of projection and persists regardless of whether the domains are modeled jointly or independently, it is better explained by a genuine difference in the overall level of self-reported emotional and sexual abuse between the BRFSS and YRBSS respondent populations than by a one-sided failure of either survey’s coefficients to transport. We cannot determine from this analysis alone why that level difference exists; right-censoring and recognition lag among adolescent respondents, true generational or reporting-context differences between the two instruments, or some combination of both, are all consistent with the pattern, and this analysis does not adjudicate between them.

This uncertainty is consistent with the broader literature showing that prospective and retrospective measures of childhood maltreatment identify substantially non-overlapping groups and show only weak-to-moderate agreement (Baldwin et al., 2019; Reuben et al., 2016). Danese and Widom similarly show that objectively documented and subjectively recalled maltreatment carry distinct associations with later psychopathology (Danese & Widom, 2020). We treat this literature as reinforcing, rather than resolving, the calibration uncertainty already documented above.

Physical abuse showed a more complex result that emerged only once the chained models were tested directly. In the marginal models, physical abuse showed the closest correspondence between predicted and observed means in both directions. Once chained on each population’s own emotional and physical abuse status, discrimination improved substantially in both directions (C-statistics rising from 0.73 to 0.81 for BRFSS→YRBSS and from 0.71 to 0.78 for YRBSS→BRFSS)—the chained models rank-order relative risk considerably better than the marginal models did. At the same time, calibration-in-the-large worsened in both directions (the gap between predicted and observed means widening from −2.3 to −5.5 percentage points BRFSS→YRBSS, and from +1.9 to +6.3 percentage points YRBSS→BRFSS). We report this trade-off rather than resolve it in the manuscript’s favor: the chained physical abuse projections rank relative risk more accurately than a marginal model would, but their absolute level carries more calibration uncertainty than the marginal model’s did.

What this analysis establishes, in total, is that none of the three abuse domain projections in Table 4 and Table 5 should be treated as calibrated point estimates without qualification, and that neither donor source’s projections should be treated as the presumptively more accurate anchor for the NSCH application. Readers should treat the absolute level of all three domains’ projections, from either donor source, with corresponding caution, weighing physical abuse’s improved discrimination against its now-documented calibration-in-the-large trade-off.

Conversely, when visible household stressors do accumulate, the probability of experienced abuse accelerates greatly with the probability of experiencing abuse reaching ≥90% in the highest-burden environments. This trajectory validates long-standing cumulative risk theories, but our synthetic model extends this logic by showing how household adversity acts as a direct multiplier for interpersonal maltreatment. The current raw NSCH metric, by failing to include the abuse domains that almost inevitably co-occur with high household adversity, substantially underestimates the 4+ ACE category among late adolescents. Raw NSCH data identify 3.8% of this age group as high-burden compared to 13.9% projected via BRFSS parameters and 7.4% via YRBSS parameters. Both projected figures should be read as directional evidence of substantial undercounting rather than a precise multiplier. Given the bidirectional calibration analysis above—which found that BRFSS-derived projections tend to over-predict, and YRBSS-derived projections tend to under-predict, emotional and sexual abuse specifically—neither figure should be treated as more accurate than the other for the NSCH application; both are presented as approximate evidence of substantial undercounting rather than precise estimates of its magnitude. As an external point of reference, Wildeman and colleagues’ NCANDS-based synthetic cohort estimate found that 12.5% of US children have a confirmed Child Protective Services maltreatment report by age 18 years (Wildeman et al., 2014)—a figure that, unlike our projections, reflects only substantiated cases and should be expected to fall below any measure capturing the fuller range of unsubstantiated or unreported abuse.

A second, distinct source of directional uncertainty arises from how the NSCH household adversity markers themselves are measured, and it applies to projections from both donor sources equally rather than favoring one over the other, unlike the donor-specific miscalibration described above. The NSCH markers are collected via parent proxy-report, using different question wording and, in most domains, meaningfully lower crude prevalence than the same constructs measured by adult or adolescent self-report in the donor surveys (Table 1; e.g., household violence: 4.3% NSCH vs. 20.6% BRFSS vs. 17.7% YRBSS). This gap implies that NSCH’s marker-positive households are, on average, a more severe and selected subset than the marker-positive group the donor coefficients were actually estimated on, while NSCH’s marker-negative households likely include a substantial number of families the donor instruments would themselves have classified as marker-positive. Both patterns push in the same direction: donor-derived coefficients likely understate the true conditional probability of abuse among both NSCH marker-positive and marker-negative households, since neither NSCH group corresponds cleanly to the group the coefficients were calibrated on. We cannot quantify the magnitude of this bias without an external, gold-standard measure of household adversities, but its direction is consistent: it suggests that the projected abuse probabilities reported in Table 4 and Table 5 are, if anything, conservative, and the true gap between raw NSCH measurement and the full ACE construct may be larger than the projected estimates presented here.

From a methodological standpoint, the close agreement between the full and reduced BRFSS parameter projections, which rarely varied by more than one to two percentage points across age cohorts, indicates that the association between the remaining household stressors and abuse is not highly sensitive to the omission of a single covariate (parental divorce) within the BRFSS donor sample. This is a specification stability check internal to BRFSS, however, and should not be read as validating the framework’s transportability to the NSCH focus population. The bidirectional calibration analysis indicates that such transportability is, in fact, imperfect for emotional and sexual abuse specifically. We therefore regard the parameter bridging approach as a useful and transparent method for illustrating the scale of a real measurement gap in NSCH rather than as a validated instrument for producing precise population estimates.

We caution against reading the composite zero-ACE and 4+-ACE shift in Supplementary Table S2 as a direct measure of the economic hardship item’s substantive importance. Removing one item from a summed categorical score mechanically compresses the distribution toward its floor and ceiling regardless of that item’s predictive value, and this arithmetic effect is proportionally larger for the YRBSS-derived score (an 8-item to 7-item reduction) than for BRFSS full (9 to 8 items), which is consistent with the YRBSS composite shifting by the largest margin in Table S3 despite the item’s negligible effect on YRBSS’s own domain-level probabilities. The domain-level results, which are not subject to this arithmetic effect, are the more direct evidence of the item’s actual contribution: real in the BRFSS-derived models, negligible in the YRBSS-derived models. We retain economic hardship in the primary analysis (Table 4 and Table 5) on this basis, while noting its limited empirical role specifically in the YRBSS-derived projections.

### 4.2. Conceptual Limitations

The cumulative ACE score used throughout this analysis, like the original Felitti et al. framework, treats 10 heterogeneous exposures—differing in type, severity, chronicity, and developmental timing—as equally weighted contributors to a single sum. This simplification is a limitation of the underlying construct (Anda et al., 2020), not only of our cross-survey projection method, and it compounds the transportability concerns discussed above: a projected ‘one ACE’ from a chained sexual abuse model and an observed ‘one ACE’ from a single household adversity marker are being added into the same total despite representing exposures that differ substantially in severity and likely impact. Readers should treat the resulting estimates as reflecting model-based projected risk under this simplified scoring convention, not as a graded or severity-weighted measure of childhood adversity.

This analysis is scoped to the foundational 10-item ACE construct introduced by Felitti and colleagues and does not address how the field’s understanding of childhood adversity has evolved since. Finkelhor et al. (2015) have made an evidence-based case for expanding the original item set to capture additional forms of adversity. The World Health Organization’s ACE-International Questionnaire (WHO, 2020) broadens the framework further to include community- and societal-level adversities alongside the household-level exposures considered here. Additionally, a separate body of work (Han et al., 2023) has argued that measuring only risk factors is incomplete, and that protective and resilience factors should be assessed alongside them. None of these developments change the measurement gap this paper identifies within the original 10-item construct as it is currently operationalized in pediatric surveillance, but readers should not extend our findings to these broader or strengths-based frameworks, which raise distinct measurement questions of their own.

### 4.3. Public Health and Policy Implications

The public health and clinical implications of this systematic underestimation are substantial. Because the raw NSCH instrument misclassifies a high volume of abused children within the zero-ACE reference category, subsequent epidemiological analyses linking the raw NSCH index to pediatric health, behavioral, or educational outcomes will suffer from attenuated effect sizes due to contaminated reference groups. Public health frameworks and clinical screening protocols must adjust for this bias, operating under the explicit assumption that the absence of observable household adversity does not guarantee a child’s safety from direct abuse. These same gaps extend beyond individual misclassification. Pediatric screening tools and surveillance systems that rely on the NSCH’s household adversity-only index will misclassify children who have experienced direct abuse but live in structurally stable households as ‘zero-ACE,’ understating their risk and potentially excluding them from indicated services. At the population level, national and state-level ACE-burden estimates derived from the NSCH alone likely understate the true prevalence of high-adversity childhoods, which has implications for how public health resources are allocated across jurisdictions. Because the magnitude of this undercounting may vary with local reporting norms and survey administration, comparisons across states or over time using the raw NSCH index should be interpreted cautiously, as apparent differences may partly reflect measurement artifact rather than true differences in childhood adversity.

## 5. Conclusions

In conclusion, this study demonstrates that evaluating pediatric adversity without measures of direct abuse compromises both the content and construct validity of the ACE score, transforming a comprehensive index, the ACE score, into a restricted measure of household level socio-environmental adversity risk. While the NSCH remains an essential tool for monitoring child well-being, researchers and policymakers must acknowledge its structural boundaries and avoid conflating household instability with the complete spectrum of childhood adversity. Because the NSCH relies entirely on parent-proxy reporting, a modality where the direct reporting of child victimization is structurally unfeasible and inherently prone to severe underestimation, expecting a primary surveillance survey of guardians to accurately capture direct abuse seems unrealistic. Consequently, the parameter-bridging methodology implemented here might not be viewed as a temporary workaround, but rather as a pragmatic, scalable corrective framework required to estimate a latent trauma footprint that cannot be directly measured at the source. Recognizing these systemic reporting boundaries and adopting rigorous cross-survey projection models may be useful for aligning public health resources, clinical screening protocols, and prevention strategies with the true scale of pediatric vulnerability in the United States.

## Figures and Tables

**Table 1:** Survey question wording for adverse childhood experiences and crude prevalence across the National Survey on Children’s Health (NSCH), Youth Risk Behavior Surveillance System (YRBSS), and Behavioral Risk Factor Surveillance System (BRFSS).

ACE Component	Survey Question(s)	Prevalence, %
Emotional abuse		
NSCH (ages 0–17 years)	Not measured.	N/A
NSCH (ages 14–17 years)	Not measured.	N/A
YRBSS (ages 14–17 years)	During your life, how often has a parent or other adult in your home insulted you or put you down?	34.4 (32.5, 36.3)
BRFSS (ages 18–49 years)	Now, looking back before you were 18 years of age, how often did a parent or adult in your home ever swear at you, insult you, or put you down?	45.0 (44.0, 46.0)
Physical abuse		
NSCH (ages 0–17 years)	Not measured.	N/A
NSCH (ages 14–17 years)	Not measured.	N/A
YRBSS (ages 14–17 years)	During your life, how often has a parent or other adult in your home hit, beat, kicked, or physically hurt you in any way?	32.1 (30.0, 34.3)
BRFSS (ages 18–49 years)	Now, looking back before you were 18 years of age, not including spanking, how often did a parent or adult in your home ever hit, beat, kick, or physically hurt you in any way?	29.8 (28.9, 30.8)
Sexual abuse		
NSCH (ages 0–17 years)	Not measured.	N/A
NSCH (ages 14–17 years)	Not measured.	N/A
YRBSS (ages 14–17 years)	Has an adult or person at least 5 years older than you ever forced you to do sexual things that you did not want to do? (Count such things as kissing, touching, or being made to have sexual intercourse.)	7.0 (6.2, 8.0)
BRFSS (ages 18–49 years)	Now, looking back before you were 18 years of age, … How often did anyone at least 5 years older than you or an adult, ever touch you sexually? How often did anyone at least 5 years older than you or an adult, try to make you touch them sexually? How often did anyone at least 5 years older than you or an adult, force you to have sex?	16.7 (16.0, 17.5)
Economic Hardship (Neglect)		
NSCH (ages 0–17 years)	Since this child was born, how often has it been very hard to cover the basics, like food or housing, on your family’s income?	14.3 (13.8, 14.8)
NSCH (ages 14–17 years)		15.2 (14.3, 16.1)
YRBSS (ages 14–17 years)	During your life, how often has there been an adult in your household who tried hard to make sure your basic needs were met, such as looking after your safety and making sure you had clean clothes and enough to eat?	8.3 (7.3, 9.3)
BRFSS (ages 18–49 years)	Now, looking back before you were 18 years of age, for how much of your childhood was there an adult in your household who tried hard to make sure your basic needs were met?	8.1 (7.6, 8.7)
HH Incarceration		
NSCH (ages 0–17 years)	To the best of your knowledge, has this child EVER experienced [a] parent or guardian who served time in jail or prison?	5.5 (5.3, 5.8)
NSCH (ages 14–17 years)		8.1 (7.5, 8.8)
YRBSS (ages 14–17 years)	Have you ever been separated from a parent or guardian because they went to jail, prison, or a detention center?	13.7 (12.4, 15.2)
BRFSS (ages 18–49 years)	Now, looking back before you were 18 years of age, did you live with anyone who served time or was sentenced to serve time in a prison, jail, or other correctional facility?	14.5 (13.8, 15.3)
HH Violence		
NSCH (ages 0–17 years)	To the best of your knowledge, has this child EVER experienced [a time when they] saw or heard parents or adults slap, hit, kick, punch one another in the home?	4.3 (4.1, 4.6)
NSCH (ages 14–17 years)		6.7 (6.1, 7.4)
YRBSS (ages 14–17 years)	During your life, how often have your parents or other adults in your home slapped, hit, kicked, punched, or beat each other up?	17.7 (16.6, 18.9)
BRFSS (ages 18–49 years)	Now, looking back before you were 18 years of age, how often did your parents or adults in your home ever slap, hit, kick, punch or beat each other up?	20.6 (19.8, 21.5)
HH Subst. Abuse		
NSCH (ages 0–17 years)	To the best of your knowledge, has this child EVER lived with anyone who had a problem with alcohol or drugs?	7.5 (7.2, 7.8)
NSCH (ages 14–17 years)		12.1 (11.4, 12.9)
YRBSS (ages 14–17 years)	Have you ever lived with a parent or guardian who was having a problem with alcohol or drug use?	24.7 (22.7, 26.9)
BRFSS (ages 18–49 years)	Now, looking back before you were 18 years of age, … Did you live with anyone who was a problem drinker or alcoholic? Did you live with anyone who used illegal street drugs or who abused prescription medication?	11.6 (11.0, 12.2)
HH Mental Health		
NSCH (ages 0–17 years)	To the best of your knowledge, has this child EVER lived with anyone who was mentally ill, suicidal, or severely depressed?	7.9 (7.6, 8.1)
NSCH (ages 14–17 years)		11.3 (10.7, 12.1)
YRBSS (ages 14–17 years)	Have you ever lived with a parent or guardian who had severe depression, anxiety, or another mental illness, or was suicidal?	28.6 (26.4, 30.9)
BRFSS (ages 18–49 years)	Now, looking back before you were 18 years of age, did you live with anyone who was depressed, mentally ill, or suicidal?	28.0 (27.1, 28.9)
Divorce/Separation		
NSCH (ages 0–17 years)	To the best of your knowledge, has this child EVER experienced [a time when a] parent or guardian divorced or separated?	20.3 (19.8, 20.8)
NSCH (ages 14–17 years)		30.7 (29.6, 31.8)
YRBSS (ages 14–17 years)	Not measured.	N/A
BRFSS (ages 18–49 years)	Now, looking back before you were 18 years of age, were your parents separated or divorced?	40.7 (39.7, 41.8)

Note: HH = household. Prevalence values reflect those estimated for the age group indicated in parentheses in the first column. 95% confidence interval reported in parentheses adjacent to prevalence estimate.

**Table 2:** a. Bivariate relationships between ACE categories observed among adults aged 18–49 years, BRFSS 2023–24. b. Bivariate relationships between ACE categories observed among adolescents aged 14–17 years, YRBSS 2023.

**a. Adults 18–49 Years, BRFSS 2023–24**									
	**Economic Hardship (Neglect)**	**HH** **Subst. Abuse**	**HH** **Incarceration**	**Sexual** **Abuse**	**HH** **Violence**	**HH** **Mental Health**	**Physical** **Abuse**	**Divorce/Separation**	**Emotional** **Abuse**
Economic Hardship (Neglect)	8.1 (7.6, 8.7)	31.5 (28.5, 34.7)	31.4 (28.2, 34.8)	39.6 (36.1, 43.1)	51.2 (47.6, 54.8)	52.0 (48.4, 55.6)	63.5 (60.0, 66.9)	61.0 (57.5, 64.5)	72.3 (68.9, 75.4)
HH Subst. Abuse	22.2 (20.0, 24.4)	11.6 (11.0, 12.2)	57.2 (54.5, 59.9)	40.7 (38.0, 43.4)	57.8 (55.0, 60.5)	71.1 (68.4, 73.6)	58.2 (55.4, 61.0)	69.2 (66.6, 71.7)	82.2 (79.9, 84.3)
HH Incarceration	17.6 (15.7, 19.6)	45.6 (42.9, 48.2)	14.6 (13.8, 15.3)	36.9 (34.2, 39.6)	49.9 (47.2, 52.6)	57.7 (55.0, 60.4)	51.1 (48.4, 53.8)	67.7 (65.2, 70.2)	73.2 (70.8, 75.5)
Sexual Abuse	19.2 (17.4, 21.2)	28.2 (26.0, 30.4)	32.1 (29.7, 34.6)	16.7 (16.0, 17.5)	45.4 (42.8, 48.0)	54.4 (51.8, 57.0)	60.7 (58.2, 63.2)	57.4 (54.7, 59.9)	75.5 (73.3, 77.6)
HH Violence	20.2 (18.5, 22.0)	32.4 (30.5, 34.5)	35.2 (33.1, 37.4)	36.8 (34.7, 39.1)	20.6 (19.8, 21.5)	55.0 (52.7, 57.2)	68.3 (66.3, 70.3)	61.3 (59.1, 63.5)	80.2 (78.3, 82.0)
HH Mental Health	15.1 (13.8, 16.5)	29.4 (27.8, 31.1)	30.0 (28.3, 31.7)	32.5 (30.7, 34.4)	40.5 (38.7, 42.3)	28.0 (27.1, 28.9)	50.1 (48.2, 51.9)	55.8 (54.0, 57.6)	75.9 (74.4, 77.4)
Physical Abuse	17.3 (15.9, 18.8)	22.6 (21.2, 24.1)	24.9 (23.3, 26.6)	34.1 (32.2, 35.9)	47.2 (45.3, 49.1)	47.0 (45.1, 48.9)	29.8 (28.9, 30.8)	51.6 (49.7, 53.5)	78.2 (76.6, 79.8)
Divorce/Separation	12.2 (11.2, 13.3)	19.7 (18.5, 20.9)	24.2 (22.8, 25.6)	23.6 (22.2, 25.0)	31.0 (29.6, 32.5)	38.4 (36.8, 39.9)	37.8 (36.2, 39.4)	40.7 (39.7, 41.8)	54.9 (53.3, 56.6)
Emotional Abuse	13.1 (12.1, 14.1)	21.2 (20.0, 22.3)	23.7 (22.4, 25.0)	28.1 (26.7, 29.5)	36.8 (35.3, 38.3)	47.2 (45.7, 48.8)	51.8 (50.3, 53.4)	49.7 (48.1, 51.3)	45.0 (44.0, 46.1)
**b. Adolescents Aged 14–17 Years, YRBSS 2023**									
	**Sexual** **Abuse**	**Economic Hardship (Neglect)**	**HH** **Incarceration**	**HH** **Violence**		**HH** **Subst. Abuse**	**HH** **Mental Health**	**Physical** **Abuse**	**Emotional Abuse**
Sexual Abuse	7.0 (6.2, 8.0)	12.4 (9.6, 16.0)	30.3 (24.3, 37.1)	46.9 (42.3, 51.7)		55.7 (47.0, 64.0)	62.0 (54.3, 69.2)	63.5 (57.4, 69.2)	71.9 (65.2, 77.8)
Economic Hardship (Neglect)	10.5 (8.3, 13.2)	8.3 (7.3, 9.3)	17.6 (14.8, 20.7)	21.8 (18.3, 25.7)		26.5 (21.4, 32.3)	24.0 (20.8, 27.5)	32.2 (26.1, 38.9)	33.5 (28.0, 39.4)
HH Incarceration	15.5 (11.9, 20.0)	10.6 (8.7, 12.8)	13.7 (12.4, 15.2)	44.1 (40.3, 48.0)		67.2 (62.2, 71.8)	56.4 (51.2, 61.4)	48.9 (44.4, 53.5)	51.6 (46.5, 56.7)
HH Violence	18.6 (15.9, 21.6)	10.2 (8.5, 12.2)	34.1 (31.0, 37.5)	17.7 (16.6, 18.9)		55.3 (50.9, 59.5)	54.8 (50.8, 58.7)	71.1 (67.4, 74.5)	68.0 (64.1, 71.6)
HH Subst. Abuse	15.8 (13.1, 18.9)	8.9 (7.6, 10.4)	37.3 (34.3, 40.3)	39.6 (37.1, 42.2)		24.8 (22.6, 26.9)	64.4 (60.4, 68.2)	50.4 (47.0, 53.8)	59.1 (55.4, 62.7)
HH Mental Health	15.3 (12.8, 18.1)	7.0 (5.9, 8.2)	27.1 (24.2, 30.1)	34.0 (31.5, 36.6)		55.8 (51.8, 59.6)	28.6 (26.4, 30.9)	46.5 (42.9, 50.1)	56.6 (53.0, 60.1)
Physical Abuse	13.9 (11.9, 16.2)	8.3 (6.9, 10.0)	20.9 (18.2, 23.9)	39.3 (36.2, 42.4)		38.8 (34.6, 43.2)	41.4 (37.0, 45.8)	32.1 (30.0, 34.3)	66.2 (62.9, 69.3)
Emotional Abuse	14.7 (12.5, 17.3)	8.1 (7.0, 9.3)	20.6 (18.0, 23.4)	35.1 (32.7, 37.5)		42.5 (38.7, 46.5)	47.0 (43.2, 50.8)	61.8 (58.8, 64.7)	34.4 (32.5, 36.3)

Note. In Table 2a, ACE categories are ordered left-to-right/top-to-bottom based on the crude ACE prevalence (%) observed among adults aged 18–49 years in the pooled 2023–24 BRFSS shown as the diagonal values. In Table 2b, ACE categories are ordered left-to-right/top-to-bottom based on the crude ACE prevalence (%) observed among adolescents aged 14–17 years in the 2023 YRBSS shown as the diagonal values. Off-diagonal values reflect the conditional probability P(column ACE|row ACE). For example, among adolescents reporting household substance abuse, 64.4% also reported living in a household with a parent or guardian who had severe depression, anxiety, or another mental illness or was suicidal.

**Table 3:** a. Prevalence of one or more, two or more, or all three types of abuse conditional on the presence of neglect and forms of household adversity observed among adults aged 18–49 years, BRFSS 2023–24. b. Prevalence of one or more, two or more, or all three types of abuse conditional on the presence of neglect and forms of household adversity observed among adolescents aged 14–17 years, YRBSS 2023.

**a. Adults Aged 18–49 Years, BRFSS 2023–24**				
	**Abuse Domain** **(Emotional, Physical, Sexual)**			
	**None**	**At Least 1 Form**	**At Least 2 Forms**	**All 3 Forms**
Overall	45.4% (44.4, 46.5)	54.6% (53.5, 55.6)	38.1% (36.9, 39.3)	9.1% (8.5, 9.7)
Economic Hardship (Neglect)	21.5 (18.6, 24.7)	78.5 (75.3, 81.4)	74.6 (71.0, 77.9)	33.6 (30.3, 37.1)
HH Incarceration	18.2 (16.2, 20.4)	81.8 (79.6, 83.8)	75.0 (72.2, 77.7)	24.7 (22.4, 27.2)
HH Violence	10.5 (9.3, 11.9)	89.5 (88.1, 90.7)	86.5 (84.8.88.1)	28.3 (26.3, 30.5)
HH Subst. Abuse	12.1 (10.2, 14.3)	87.9 (85.7, 89.8)	84.2 (81.5, 86.6)	28.8 (26.4, 31.3)
HH Mental Health	17.6 (16.3, 19.0)	82.4 (81.0, 83.7)	75.5 (73.6, 77.3)	22.0 (20.3, 23.7)
Divorce/Separation	35.1 (33.6, 36.8)	64.9 (63.2, 66.4)	51.7 (49.8, 53.6)	13.8 (12.7, 15.0)
HH Adversity				
0 of 5 (41.9%)	66.0 (64.4, 67.5)	34.0 (32.5, 35.6)	13.2 (11.9, 14.6)	1.2 (1.0, 1.6)
1 of 5 (28.4%)	47.7 (45.7, 49.6)	52.3 (50.4, 54.3)	32.6 (30.5, 34.9)	6.0 (5.0, 7.2)
2 of 5 (13.1%)	22.5 (20.4, 24.8)	77.5 (75.2, 79.6)	66.0 (62.8, 69.0)	13.5 (11.6, 15.7)
3 of 5 (8.0%)	13.5 (11.3, 16.1)	86.5 (83.9, 88.7)	81.7 (78.2, 84.7)	22.9 (19.9, 26.2)
4 of 5 (5.5%)	5.4 (3.9, 7.5)	94.6 (92.5, 96.1)	93.3 (90.9, 95.2)	36.2 (32.0, 40.7)
5 of 5 (3.1%)	4.3 (1.9, 9.3)	95.7 (90.7, 98.1)	95.0 (89.2, 97.8)	46.8 (41.3, 52.4)
**b. Adolescents Aged 14–17 Years, YRBSS 2023**				
	**Abuse Domain** **(Emotional, Physical, Sexual)**			
	**None**	**At Least 1 Form**	**At Least 2 Forms**	**All 3 Forms**
Overall	53.3% (51.2, 55.3)	46.7% (44.7, 48.8)	30.1% (27.8, 32.4)	3.9% (3.3, 4.7)
Economic Hardship (Neglect)	54.1 (47.9, 60.3)	45.9 (39.7, 52.1)	30.5 (24.1, 37.7)	6.6 (4.9, 8.9)
HH Incarceration	33.7 (28.7, 39.1)	66.3 (60.9, 71.3)	53.7 (47.2, 60.2)	10.6 (7.6, 14.7)
HH Violence	15.6 (13.2, 18.3)	84.4 (81.7, 86.8)	79.1 (75.4, 82.3)	14.3 (11.7, 17.4)
HH Subst. Abuse	28.7 (25.9, 31.6)	71.3 (68.4, 74.1)	60.3 (55.9, 64.5)	10.5 (8.1, 13.4)
HH Mental Health	31.7 (28.8, 34.8)	68.3 (65.2, 71.2)	55.9 (51.5, 60.1)	9.9 (7.8, 12.5)
HH Adversity				
0 of 4 (54.6%)	70.0 (67.0, 72.8)	30.0 (27.2, 33.0)	12.3 (10.6, 14.3)	<1 (0.4, 1.0)
1 of 4 (21.4%)	42.8 (39.4, 46.2)	57.2 (53.8, 60.6)	40.5 (35.5, 45.7)	3.0 (2.2, 4.0)
2 of 4 (12.5%)	30.9 (27.5, 34.5)	69.1 (65.5, 72.5)	54.8 (49.3, 60.1)	8.3 (6.2, 11.0)
3 of 4 (7.7%)	21.5 (17.8, 25.8)	78.5 (74.2, 82.2)	70.9 (65.8, 75.5)	12.5 (8.8, 17.4)
4 of 4 (3.8%)	11.1 (7.4, 16.3)	88.9 (83.7, 92.6)	86.0 (79.1, 90.9)	25.3 (17.3, 35.3)

Note. HH = household. The HH adversity domain score reflects the simple summation of the presence (=1) or absence (=0) of incarceration, domestic violence, substance abuse, mental health, divorce/separation. Table values reflect the conditional probability P(column value|row ACE). HH = household. The HH adversity domain score reflects the simple summation of the presence (=1) or absence (=0) of incarceration, domestic violence, substance abuse, mental health. The YRBSS did not ask whether the adolescent EVER experienced [a time when] a parent or guardian divorced or separated. Table values reflect the conditional probability P(column value|row ACE).

**Table 4:** Summary emotional, physical and sexual abuse prevalence estimate projections by age group for children aged <18 years.

	Emotional Abuse			Physical Abuse			Sexual Abuse		
Age Group	Imputed from BRFSS (Full)	Imputed from BRFSS (Reduced)	Imputed from YRBSS	Imputed from BRFSS (Full)	Imputed from BRFSS (Reduced)	Imputed from YRBSS	Imputed from BRFSS (Full)	Imputed from BRFSS (Reduced)	Imputed from YRBSS
<12 months	29.2 (25.4, 33.1)	29.5 (25.9, 33.1)	22.2 (18.0, 26.4)	18.0 (14.3, 21.6)	18.0 (14.5, 21.4)	20.4 (16.0, 24.8)	7.9 (5.5, 10.4)	8.7 (6.0, 11.4)	3.1 (1.4, 4.8)
1–2 years	29.7 (26.9, 32.4)	30.2 (27.5, 32.8)	22.8 (19.7, 25.9)	18.7 (16.0, 21.4)	18.8 (16.4, 21.2)	20.9 (17.6, 24.2)	8.7 (7.2, 10.2)	9.4 (7.8, 11.0)	3.4 (2.3, 4.5)
3–4 years	30.9 (28.2, 33.7)	31.5 (29.2, 33.7)	23.5 (20.5, 26.6)	19.7 (17.3, 22.0)	19.9 (17.8, 22.1)	21.7 (18.1, 25.3)	9.5 (7.7, 11.3)	9.9 (8.4, 11.5)	3.5 (2.4, 4.7)
5–7 years	32.3 (30.1, 34.6)	32.9 (30.6, 35.3)	24.3 (21.6, 27.0)	20.5 (18.2, 22.7)	20.7 (18.8, 22.6)	22.4 (19.4, 25.4)	10.1 (8.6, 11.7)	10.6 (9.1, 12.1)	3.7 (2.8, 4.7)
8–9 years	33.8 (31.1, 36.4)	34.4 (31.6, 37.2)	25.1 (22.2, 28.0)	21.6 (19.0, 24.2)	21.8 (19.4, 24.2)	22.9 (19.4, 26.4)	11.0 (9.0, 13.0)	11.4 (9.7, 13.2)	4.2 (3.0, 5.4)
10–13 years	34.2 (32.1, 36.2)	34.5 (32.6, 36.5)	25.5 (23.1, 27.8)	22.0 (20.1, 24.0)	22.1 (20.4, 23.7)	23.5 (20.7, 26.3)	11.5 (10.2, 12.7)	11.7 (10.3, 13.1)	4.1 (3.3, 5.0)
14–17 years	36.1 (34.0, 38.2)	36.2 (34.4, 38.0)	26.7 (24.3, 29.0)	23.6 (21.8, 25.4)	23.5 (21.8, 25.3)	24.7 (22.1, 27.4)	12.6 (11.4, 13.9)	12.6 (11.1, 14.2)	4.7 (3.7, 5.7)

Note. Emotional, physical, and sexual abuse are imputed via chained equations (emotional abuse first, then physical abuse conditional on imputed emotional abuse status, then sexual abuse conditional on both). **Imputed from BRFSS (Full)** reflects projection of parameter relationships from model, including five household adversity stressors plus economic hardship (neglect) obtained from BRFSS data. **Imputed from BRFSS (Reduced)** reflects projection of parameter relationships from model, including four of the five household adversity stressors, divorce/separation excluded, plus economic hardship obtained from BRFSS data. **Imputed from YRBSS** reflects projection of parameter relationships from model, including four household adversity stressors (divorce/separation was not measured) plus economic hardship from YRBSS data. Table values are reported as percentages with 95% simulation intervals in parentheses reflecting donor parameter uncertainty and Bernoulli draw variance only, and do not include transport, misspecification, or measurement-differential uncertainty.

**Table 5:** Summary ACE score prevalence for a score of zero (0) and of 4 or more (4+) ACEs by age group for children aged <18 years.

	HH Adversity + Economic Hardship NSCH (Raw)		HH Adversity + Economic Hardship + Abuse NSCH Plus Abuse Imputed from BRFSS (Full)		HH Adversity + Economic Hardship + Abuse NSCH Plus Abuse Imputed from BRFSS (Reduced)		HH Adversity + Economic Hardship + Abuse NSCH Plus Abuse Imputed from YRBSS (Reduced)	
Age Group	0 ACEs	4+ ACEs	0 ACEs	4+ ACEs	0 ACEs	4+ ACEs	0 ACEs	4+ ACEs
<12 months	93.9 (92.5–95.0)	0.2 (0.0–0.6)	53.4 (49.3, 57.5)	2.0 (0.9, 3.1)	52.8 (48.8, 56.8)	2.1 (0.9, 3.2)	56.6 (51.6, 61.5)	0.7 (0.3, 1.2)
1–2 years	88.3 (86.9–89.6)	0.6 (0.4–0.9)	51.1 (48.5, 53.6)	3.7 (2.7, 4.7)	50.3 (47.7, 52.9)	3.7 (2.8, 4.6)	55.2 (52.2, 58.1)	1.7 (1.0, 2.3)
3–4 years	82.9 (81.4–84.3)	1.0 (0.7–1.4)	48.3 (45.6, 50.9)	5.8 (4.7, 6.8)	47.3 (45.0, 49.6)	5.7 (4.7, 6.7)	53.7 (50.7, 56.8)	2.8 (2.1, 3.5)
5–7 years	78.1 (76.8–79.4)	1.7 (1.4–2.1)	44.6 (42.5, 46.7)	7.4 (6.4, 8.3)	43.8 (41.8, 45.8)	7.3 (6.4, 8.3)	51.6 (49.1, 54.0)	3.8 (3.0, 4.5)
8–9 years	72.2 (70.5–73.8)	2.6 (2.1–3.2)	42.5 (39.9, 45.2)	9.8 (8.3, 11.2)	41.6 (39.0, 44.1)	9.6 (8.3, 11.0)	50.2 (47.2, 53.3)	5.2 (4.2, 6.3)
10–13 years	67.0 (65.8–68.2)	3.1 (2.7–3.5)	39.4 (37.6, 41.3)	10.7 (9.7, 11.7)	38.7 (37.0, 40.5)	10.7 (9.7, 11.6)	49.2 (46.7, 51.7)	5.7 (5.0, 6.4)
14–17 years	61.4 (60.2–62.5)	3.8 (3.4–4.2)	36.2 (34.7, 37.7)	13.9 (12.8, 14.9)	35.6 (34.0, 37.2)	13.6 (12.6, 14.6)	46.3 (44.1, 48.5)	7.4 (6.6, 8.1)

Note. Summary of ACE scores based on imputed emotional, physical, and sexual abuse values via chained equations (emotional abuse first, then physical abuse conditional on imputed emotional abuse status, then sexual abuse conditional on both) and reported household adversity. See notes for Table 4.

## Data Availability

All data used for this analysis are publicly available for download from https://www.cdc.gov/brfss (accessed on 6 June 2026), https://www.cdc.gov/yrbs (accessed on 6 June 2026), and https://mchb.hrsa.gov/data-research/national-survey-childrens-health (accessed on 6 June 2026). Analytic code is available from the authors on request.

## Supplementary Materials

The following supporting information can be downloaded at: https://www.mdpi.com/article/10.3390/bs16091652/s1.
