# Supplementary material for: Beyond Household Adversities: Synthetic Imputation of Emotional, Physical and Sexual Abuse for Children Aged <18 Years in the National Survey on Children’s Health (NSCH) Through Parameter Bridging from the Behavioral Risk Factor Surveillance System (BRFSS) and Youth Risk Behavior Surveillance System (YRBSS)
Source: Behav Sci (Basel). 2026 Sep 15;16(9):1652. doi: 10.3390/bs16091652 (PMC13603256; doi:10.3390/bs16091652)
Supplement: Supplementary file 1 [file behavsci-16-01652-s001.zip › behavsci-4483395-supplementary.pdf]

Supplementary Table S1. BRFSS State Added ACE Module Inclusion Analysis Sample

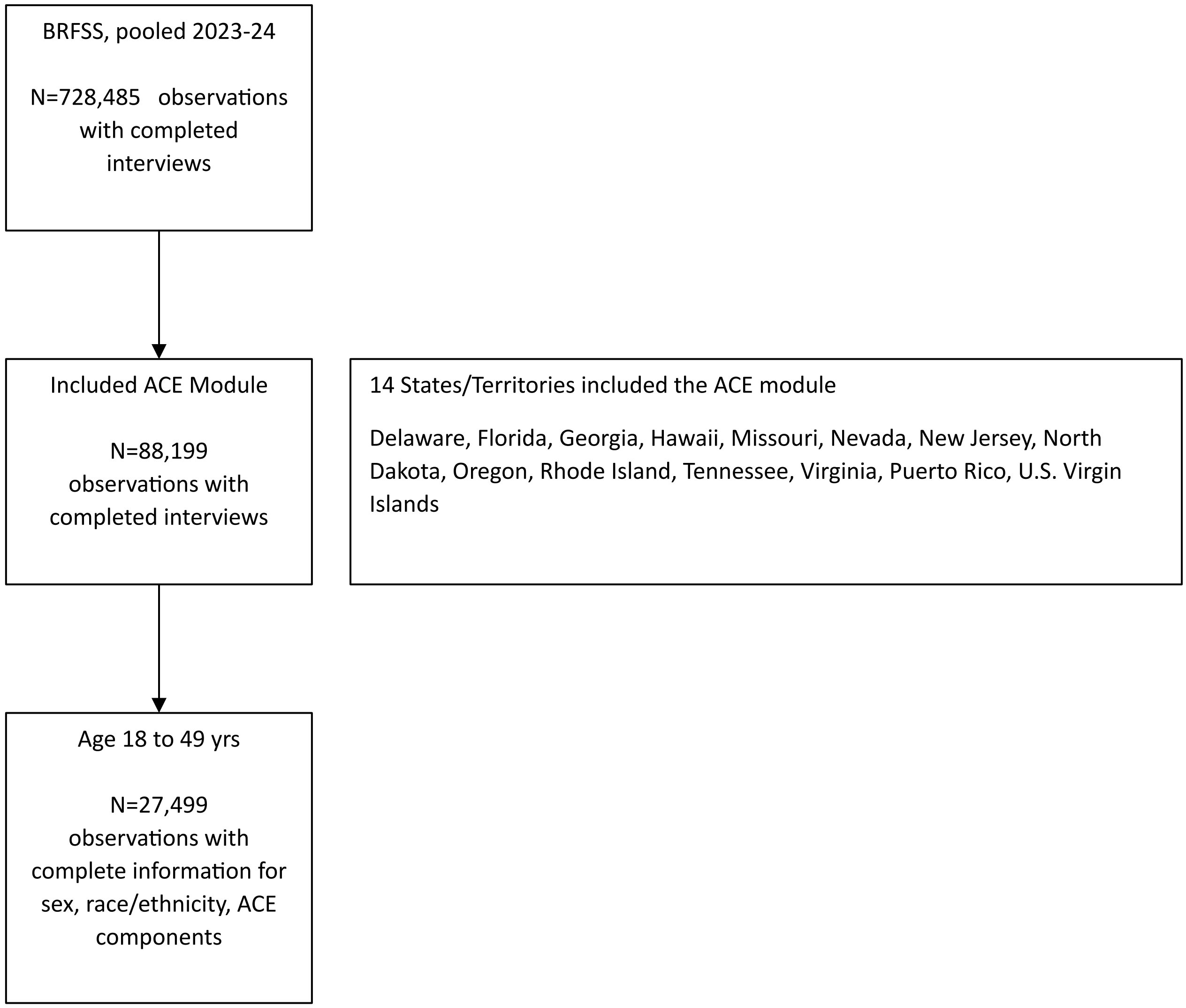

Supplementary Table S2. Sensitivity Analysis: Economic Hardship Item Included vs. Excluded

Point-estimate percentages from donor models and NSCH projections re-estimated with the economic-hardship item removed entirely from both the abuse-domain covariate sets and the composite ACE score (BRFSS Full, BRFSS Reduced, YRBSS, versus the primary specifications in Table 5). "With" columns reproduce the primary chained results from Table 4/5; "Without" columns are this sensitivity analysis. 95% intervals are omitted here for compactness.

BRFSS (Full)

	**Emotional Abuse**		**Physical Abuse**		**Sexual Abuse**		**0 ACEs**		**4+ ACEs**	
**Age**	**With**	**Without**	**With**	**Without**	**With**	**Without**	**With**	**Without**	**With**	**Without**
**< 12 Months**	29.2%	27.0%	18.0%	15.6%	7.9%	7.2%	53.4%	60.8%	2.0%	0.9%
**1–2 Years**	29.7	27.9	18.7	16.7	8.7	8.2	51.1	57.0	3.7	2.2
**3–4 Years**	30.9	29.2	19.7	17.8	9.5	8.8	48.3	53.3	5.8	3.8
**5–7 Years**	32.3	30.6	20.5	18.5	10.1	9.4	44.6	50.1	7.4	5.2
**8–9 Years**	33.8	32.2	21.6	19.6	11.0	10.2	42.5	46.5	9.8	7.1
**10–13 Years**	34.2	32.7	22.0	20.1	11.5	10.7	39.4	43.2	10.7	8.5
**14–17 Years**	36.1	34.5	23.6	21.5	12.6	11.8	36.2	39.6	13.9	11.1

BRFSS (Reduced)

	**Emotional Abuse**		**Physical Abuse**		**Sexual Abuse**		**0 ACEs**		**4+ ACEs**	
**Age**	**With**	**Without**	**With**	**Without**	**With**	**Without**	**With**	**Without**	**With**	**Without**
**< 12 Months**	29.5	28.1	18.0	16.4	8.7	8.1	52.8	59.2	2.1	0.9
**1–2 Years**	30.2	29.0	18.8	17.5	9.4	8.5	50.3	55.7	3.7	2.2
**3–4 Years**	31.5	30.2	19.9	18.1	9.9	9.1	47.3	52.1	5.7	3.7
**5–7 Years**	32.9	31.0	20.7	19.0	10.6	9.8	43.8	49.1	7.3	5.2
**8–9 Years**	34.4	32.8	21.8	19.8	11.4	10.4	41.6	45.6	9.6	7.0
**10–13 Years**	34.5	33.1	22.1	20.4	11.7	10.8	38.7	42.2	10.7	8.4
**14–17 Years**	36.2	34.7	23.5	21.8	12.6	11.7	35.6	38.7	13.6	10.9

YRBSS

	Emotional Abuse		Physical Abuse		Sexual Abuse		0 ACEs		4+ ACEs	
**Age**	**With**	**Without**	**With**	**Without**	**With**	**Without**	**With**	**Without**	**With**	**Without**
**< 12 Months**	22.2	21.9	20.4	20.4	3.1	3.0	56.6	64.9	0.7	0.5
**1–2 Years**	22.8	22.7	20.9	21.1	3.4	3.2	55.2	62.5	1.7	1.1
**3–4 Years**	23.5	23.3	21.7	21.8	3.5	3.6	53.7	61.0	2.8	2.0
**5–7 Years**	24.3	24.2	22.4	22.7	3.7	3.6	51.6	58.8	3.8	2.9
**8–9 Years**	25.1	25.0	22.9	23.3	4.2	4.0	50.2	56.4	5.2	4.1
**10–13 Years**	25.5	25.5	23.5	23.9	4.1	4.0	49.2	55.0	5.7	4.7
**14–17 Years**	26.7	26.7	24.7	25.1	4.7	4.4	46.3	52.0	7.4	6.0

Supplementary Table S3. Donor Model Coefficients, Standard Errors, and Odds Ratios

Full coefficient output for the nine donor models underlying the final, chained Table 4 and Table 5 projections. The *Chained input* rows indicate covariates that are the previously imputed abuse-domain status within the chained-equations sequence, not observed predictors. Reference category for race/ethnicity is White, non-Hispanic. Coefficients, standard errors, and confidence intervals are on the log-odds scale as reported by Stata's design-based (svy) logistic regression; odds ratios and their confidence intervals are exponentiated from the same estimates.

BRFSS Full — Emotional Abuse

Predictor	Coef. (log-odds)	SE	Odds Ratio	OR 95% CI	Coef. 95% CI
Household Mental Illness	1.3571	0.0572	3.88	(3.47, 4.35)	(1.245, 1.469)
Household Substance Abuse	0.6870	0.0992	1.99	(1.64, 2.41)	(0.493, 0.881)
Household Incarceration	0.3992	0.0854	1.49	(1.26, 1.76)	(0.232, 0.567)
Household Violence	1.4749	0.0707	4.37	(3.81, 5.02)	(1.336, 1.613)
Divorce/Separation	0.2052	0.0529	1.23	(1.11, 1.36)	(0.102, 0.309)
Economic Hardship	0.7266	0.1026	2.07	(1.69, 2.53)	(0.526, 0.928)
Female	-0.0171	0.0503	0.98	(0.89, 1.08)	(-0.116, 0.082)
Race: Black NH (vs. White NH)	-0.0952	0.0767	0.91	(0.78, 1.06)	(-0.245, 0.055)
Race: Other NH (vs. White NH)	0.1170	0.0825	1.12	(0.96, 1.32)	(-0.045, 0.279)
Race: Hispanic (vs. White NH)	-0.6870	0.0679	0.50	(0.44, 0.57)	(-0.820, -0.554)
Constant	-0.9267	0.0438	0.40	(0.36, 0.43)	(-1.013, -0.841)

$F(10, 135660) = 157.63$, $p<0.001$

BRFSS Full — Physical Abuse

Predictor	Coef. (log-odds)	SE	Odds Ratio	OR 95% CI	Coef. 95% CI
Household Mental Illness	0.4010	0.0625	1.49	(1.32, 1.69)	(0.279, 0.523)
Household Substance Abuse	0.1302	0.0846	1.14	(0.97, 1.34)	(-0.036, 0.296)
Household Incarceration	0.0057	0.0829	1.01	(0.85, 1.18)	(-0.157, 0.168)
Household Violence	1.4519	0.0645	4.27	(3.76, 4.85)	(1.325, 1.578)
Divorce/Separation	0.0650	0.0579	1.07	(0.95, 1.20)	(-0.048, 0.178)
Economic Hardship	0.8457	0.0905	2.33	(1.95, 2.78)	(0.668, 1.023)
Emotional Abuse (chain input)	1.6689	0.0622	5.31	(4.70, 5.99)	(1.547, 1.791)
Female	-0.1583	0.0558	0.85	(0.77, 0.95)	(-0.268, -0.049)
Race: Black NH (vs. White NH)	0.3277	0.0858	1.39	(1.17, 1.64)	(0.160, 0.496)
Race: Other NH (vs. White NH)	0.3839	0.0861	1.47	(1.24, 1.74)	(0.215, 0.553)
Race: Hispanic (vs. White NH)	0.7048	0.0755	2.02	(1.75, 2.35)	(0.557, 0.853)
Constant	-2.5828	0.0643	0.08	(0.07, 0.09)	(-2.709, -2.457)

$F(11, 135659) = 196.50$, $p<0.001$

BRFSS Full — Sexual Abuse

Predictor	Coef. (log-odds)	SE	Odds Ratio	OR 95% CI	Coef. 95% CI
Household Mental Illness	0.5572	0.0767	1.75	(1.50, 2.03)	(0.407, 0.708)
Household Substance Abuse	0.3172	0.0936	1.37	(1.14, 1.65)	(0.134, 0.501)
Household Incarceration	0.5179	0.0872	1.68	(1.41, 1.99)	(0.347, 0.689)
Household Violence	0.3703	0.0844	1.45	(1.23, 1.71)	(0.205, 0.536)
Divorce/Separation	0.2654	0.0695	1.30	(1.14, 1.49)	(0.129, 0.402)
Economic Hardship	0.4102	0.0909	1.51	(1.26, 1.80)	(0.232, 0.588)
Emotional Abuse (chain input)	0.6976	0.0822	2.01	(1.71, 2.36)	(0.537, 0.859)
Physical Abuse (chain input)	0.9124	0.0758	2.49	(2.15, 2.89)	(0.764, 1.061)
Female	1.0992	0.0709	3.00	(2.61, 3.45)	(0.960, 1.238)
Race: Black NH (vs. White NH)	0.6695	0.0959	1.95	(1.62, 2.36)	(0.482, 0.857)
Race: Other NH (vs. White NH)	-0.0679	0.1020	0.93	(0.77, 1.14)	(-0.268, 0.132)
Race: Hispanic (vs. White NH)	0.1476	0.0885	1.16	(0.97, 1.38)	(-0.026, 0.321)
Constant	-3.8233	0.0854	0.02	(0.02, 0.03)	(-3.991, -3.656)

$F(12, 135658) = 126.77$, $p<0.001$

BRFSS Reduced — Emotional Abuse

Predictor	Coef. (log-odds)	SE	Odds Ratio	OR 95% CI	Coef. 95% CI
Household Mental Illness	1.3752	0.0569	3.96	(3.54, 4.42)	(1.264, 1.487)
Household Substance Abuse	0.7138	0.0988	2.04	(1.68, 2.48)	(0.520, 0.907)
Household Incarceration	0.4389	0.0844	1.55	(1.31, 1.83)	(0.273, 0.604)
Household Violence	1.5010	0.0703	4.49	(3.91, 5.15)	(1.363, 1.639)
Economic Hardship	0.7439	0.1031	2.10	(1.72, 2.58)	(0.542, 0.946)
Female	-0.0161	0.0503	0.98	(0.89, 1.09)	(-0.115, 0.082)
Race: Black NH (vs. White NH)	-0.0804	0.0761	0.92	(0.79, 1.07)	(-0.230, 0.069)
Race: Other NH (vs. White NH)	0.0977	0.0822	1.10	(0.94, 1.30)	(-0.063, 0.259)
Race: Hispanic (vs. White NH)	-0.6770	0.0679	0.51	(0.44, 0.58)	(-0.810, -0.544)
Constant	-0.8661	0.0411	0.42	(0.39, 0.46)	(-0.947, -0.786)

$F(9, 135661) = 175.49, p<0.001$

BRFSS Reduced — Physical Abuse

Predictor	Coef. (log-odds)	SE	Odds Ratio	OR 95% CI	Coef. 95% CI
Household Mental Illness	0.4068	0.0620	1.50	(1.33, 1.70)	(0.285, 0.528)
Household Substance Abuse	0.1379	0.0842	1.15	(0.97, 1.35)	(-0.027, 0.303)
Household Incarceration	0.0165	0.0831	1.02	(0.86, 1.20)	(-0.146, 0.179)
Household Violence	1.4598	0.0641	4.31	(3.80, 4.88)	(1.334, 1.585)
Economic Hardship	0.8521	0.0903	2.34	(1.96, 2.80)	(0.675, 1.029)
Emotional Abuse (chain input)	1.6711	0.0620	5.32	(4.71, 6.01)	(1.549, 1.793)
Female	-0.1583	0.0557	0.85	(0.77, 0.95)	(-0.267, -0.049)
Race: Black NH (vs. White NH)	0.3310	0.0858	1.39	(1.18, 1.65)	(0.163, 0.499)
Race: Other NH (vs. White NH)	0.3784	0.0861	1.46	(1.23, 1.73)	(0.210, 0.547)
Race: Hispanic (vs. White NH)	0.7067	0.0753	2.03	(1.75, 2.35)	(0.559, 0.854)
Constant	-2.5632	0.0620	0.08	(0.07, 0.09)	(-2.685, -2.442)

$F(10, 135660) = 209.08, p<0.001$

BRFSS Reduced — Sexual Abuse

Predictor	Coef. (log-odds)	SE	Odds Ratio	OR 95% CI	Coef. 95% CI
Household Mental Illness	0.5786	0.0760	1.78	(1.54, 2.07)	(0.430, 0.728)
Household Substance Abuse	0.3464	0.0931	1.41	(1.18, 1.70)	(0.164, 0.529)
Household Incarceration	0.5585	0.0859	1.75	(1.48, 2.07)	(0.390, 0.727)
Household Violence	0.3967	0.0838	1.49	(1.26, 1.75)	(0.233, 0.561)
Economic Hardship	0.4367	0.0912	1.55	(1.29, 1.85)	(0.258, 0.615)
Emotional Abuse (chain input)	0.7046	0.0821	2.02	(1.72, 2.38)	(0.544, 0.865)
Physical Abuse (chain input)	0.9152	0.0757	2.50	(2.15, 2.90)	(0.767, 1.064)
Female	1.0950	0.0705	2.99	(2.60, 3.43)	(0.957, 1.233)
Race: Black NH (vs. White NH)	0.6830	0.0955	1.98	(1.64, 2.39)	(0.496, 0.870)
Race: Other NH (vs. White NH)	-0.0873	0.1011	0.92	(0.75, 1.12)	(-0.285, 0.111)
Race: Hispanic (vs. White NH)	0.1491	0.0881	1.16	(0.98, 1.38)	(-0.024, 0.322)
Constant	-3.7323	0.0832	0.02	(0.02, 0.03)	(-3.895, -3.569)

F(11, 135659) = 135.54, p<0.001

YRBS — Emotional Abuse

Predictor	Coef. (log-odds)	SE	Odds Ratio	OR 95% CI	Coef. 95% CI
Household Mental Illness	0.7549	0.0883	2.13	(1.78, 2.54)	(0.579, 0.931)
Household Substance Abuse	0.7766	0.0804	2.17	(1.85, 2.55)	(0.616, 0.937)
Household Incarceration	-0.1277	0.1076	0.88	(0.71, 1.09)	(-0.342, 0.087)
Household Violence	1.3115	0.0894	3.71	(3.11, 4.44)	(1.133, 1.490)
Economic Hardship	-0.0296	0.1316	0.97	(0.75, 1.26)	(-0.292, 0.233)
Female	0.7681	0.0712	2.16	(1.87, 2.48)	(0.626, 0.910)
Race: Black NH (vs. White NH)	0.0907	0.1366	1.09	(0.83, 1.44)	(-0.182, 0.363)
Race: Other NH (vs. White NH)	0.2597	0.1117	1.30	(1.04, 1.62)	(0.037, 0.482)
Race: Hispanic (vs. White NH)	-0.0591	0.0969	0.94	(0.78, 1.14)	(-0.252, 0.134)
Constant	-1.7462	0.0590	0.17	(0.16, 0.20)	(-1.864, -1.629)

F(9, 65) = 77.76, p<0.001

YRBS — Physical Abuse

Predictor	Coef. (log-odds)	SE	Odds Ratio	OR 95% CI	Coef. 95% CI
Household Mental Illness	0.1515	0.0980	1.16	(0.96, 1.41)	(-0.044, 0.347)
Household Substance Abuse	0.2667	0.0924	1.31	(1.09, 1.57)	(0.083, 0.451)
Household Incarceration	-0.0387	0.1019	0.96	(0.79, 1.18)	(-0.242, 0.164)
Household Violence	1.5563	0.1077	4.74	(3.83, 5.88)	(1.342, 1.771)
Emotional Abuse (chain input)	1.9220	0.0789	6.83	(5.84, 8.00)	(1.765, 2.079)
Female	-0.5002	0.0730	0.61	(0.52, 0.70)	(-0.646, -0.355)
Race: Black NH (vs. White NH)	0.8421	0.1761	2.32	(1.63, 3.30)	(0.491, 1.193)
Race: Other NH (vs. White NH)	0.5923	0.1495	1.81	(1.34, 2.44)	(0.294, 0.890)
Race: Hispanic (vs. White NH)	0.4917	0.1020	1.64	(1.33, 2.00)	(0.288, 0.695)
Constant	-2.0614	0.1139	0.13	(0.10, 0.16)	(-2.288, -1.834)

F(10, 64) = 106.11, p<0.001

YRBS — Sexual Abuse

Predictor	Coef. (log-odds)	SE	Odds Ratio	OR 95% CI	Coef. 95% CI
Household Mental Illness	0.7343	0.1091	2.08	(1.68, 2.59)	(0.517, 0.952)
Household Substance Abuse	0.4705	0.1298	1.60	(1.24, 2.07)	(0.212, 0.729)
Household Incarceration	0.2454	0.1720	1.28	(0.91, 1.80)	(-0.097, 0.588)
Household Violence	0.4938	0.1028	1.64	(1.34, 2.01)	(0.289, 0.699)
Emotional Abuse (chain input)	0.7285	0.1498	2.07	(1.54, 2.79)	(0.430, 1.027)
Physical Abuse (chain input)	0.6230	0.1382	1.86	(1.42, 2.46)	(0.347, 0.899)
Female	1.3282	0.1781	3.77	(2.65, 5.38)	(0.973, 1.683)
Race: Black NH (vs. White NH)	-0.0218	0.2029	0.98	(0.65, 1.47)	(-0.426, 0.383)
Race: Other NH (vs. White NH)	0.0795	0.1813	1.08	(0.75, 1.55)	(-0.282, 0.441)
Race: Hispanic (vs. White NH)	0.4437	0.1562	1.56	(1.14, 2.13)	(0.132, 0.755)
Constant	-5.0116	0.1586	0.01	(0.00, 0.01)	(-5.328, -4.696)

F(11, 63) = 67.69, p<0.001
